# A causal inference method for athletic injuries based on quantile threshold functions and latent Gaussian DAG models

**DOI:** 10.3389/fpubh.2025.1647200

**Published:** 2025-09-10

**Authors:** Tao Xie, Yaxian Hao, Fen Xie

**Affiliations:** ^1^Department of Sports Science, Kyungil University, Gyeongsan, Republic of Korea; ^2^School of Mathematics and Computer Science, Shanxi Normal University, Taiyuan, China; ^3^Department of Data Science, City University of Hong Kong, Kowloon, Hong Kong SAR, China

**Keywords:** causal inference, athletic injury, directed acyclic graph, latent graphical model, ordinal causal effects

## Abstract

**Introduction:**

Causal inference of athletic injuries provides the critical foundations for the development of effective prevention strategies. In recent years, the directed acyclic graph model (DAG) has established itself as an indispensable tool in the study of athletic injuries.

**Methods:**

This study proposes a quantile threshold function (QTF) and integrates it with the causal inference framework within the latent DAG model for ordinal variables. This process begins by transforming continuous variables into ordinal variables to construct a DAG, which is analyzed using the latent causal inference framework to estimate ordinal causal effects (OCE).

**Results:**

Testing this approach on real-world data showed clear differences between groups (*F* > 52,000, *P* < 0.05). The analysis also revealed three direct paths and two indirect paths related to athletic injuries, based on the DAG.

**Discussion:**

We obtained the OCE by intervening on variables that directly or indirectly influence athletic injuries. DAG path analysis further elucidated the impact of causal pathways on the risk of injury. The approach proposed in this study provides novel theoretical and methodological insights into athletic injuries and serves as a crucial basis for optimizing training programs and mitigating injury risk.

## 1 Introduction

Sports science research is fundamentally causal (1, 2), focusing on the mechanisms of physical activity to optimize training and strategy. Identifying determinants of athletic or team success informs performance improvement, while in health research, physical activity interventions improve fitness and well-being. Data in sports science are often highly discrete. This characteristic can be addressed through the use of scientific methods that categorize the data into distinct levels, enabling it to be treated as ordinal variables. Ordinal variables, characterized by categorical values with an inherent ranking order, are prevalent in various research fields (3). Examples include training intensity (A, B, C) and training frequency (low, medium, high). Considering the widespread occurrence of ordinal variables in sports data, investigating methods of causal effect analysis for these variables has considerable practical significance.

Causal inference aims to uncover these underlying relationships. Epidemiological research is a key area of causal inference in sports science (4). Although some advocate causal models in injury prevention (5, 6), practical applications remain limited (7). Early studies introduced graphical causal models (6, 8), but limitations in presentation and scope constrained their impact. (9) emphasized causal reasoning in strength training, although their work focused on specific issues rather than a systematic introduction. The van Mechelen sequence of prevention (10) and the Finch TRIPP framework (11) introduce measures that are likely to reduce the future risk and/or severity of athletic injuries based on causal and mechanistic understandings. However, the development of causal knowledge represents a significant challenge. To estimate a causal effect, researchers must control all major baseline variables that could influence both exposure and outcome (12, 13). However, fulfilling these conditions in real-world settings can be exceptionally challenging. Failure to control a confounding variable can lead to inaccurate conclusions about the causal relationship between variables. Randomized controlled trials (RCTs), which are considered the gold standard for causal inference, were later proposed by researchers but are challenging to implement in sports science (14), particularly in elite sports (15). Therefore, inference of causal relationships often relies on observational studies, which are prone to selection bias. The reliance on such studies, combined with the lack of robust tools and frameworks for causal inference, has hindered the advancement of causal knowledge on sports injuries and the development of effective prevention strategies. Competitive sports training often involves a high risk of sports injuries, not only affecting the overall volume of training, but may also alter training patterns and recovery strategies (16, 17).

To address some of the issues mentioned above, recent efforts have emphasized the adoption of graphical causal models in injury prevention and advocated for greater participation in causal inference research (18). Causal diagrams, including frameworks (19), models (20, 21), causal directed acyclic graphs (DAG) (22, 23), and other types of diagrams (24, 25), serve as valuable tools for organizing ideas, guiding future research, and supporting causal inference efforts. These diagrams, particularly DAG, are of significant importance in statistical analysis. In most practical situations, an appropriate causal diagram is rarely known, so methods that can learn both a network structure and its parameters from data are required. A Bayesian network is the most commonly used method for causal graph problems. When using Bayesian methods for learning, the observed data only determine the DAG describing their joint distribution up to its Markov equivalence class (26). It is crucial that each Markov equivalence class can be uniquely represented by a completed partially directed acyclic graph(CPDAG). The learning of Bayesian networks relies fundamentally on the type of data, with existing approaches focused primarily on continuous and categorical data (27, 28). However, in the field of sports training, the variables involved often include both continuous data and ordinal data. Existing methods do not fully consider the inherent ordinal nature of the data when handling ordinal data. Therefore, specialized methods are needed to calculate the causal effects between ordinal variables while fully accounting for their ordinal characteristics. Luo et al. (29) proposed an Ordinal Structural Expectation-Maximization (OSEM) algorithm based on a latent Gaussian model. This algorithm can construct an appropriate causal graph framework for ordinal data (29), providing support for a subsequent analysis of causal effects.

Realizing the existing gaps in the theoretical and practical aspects of this field, this study proposes a quantile threshold function (QTF) that transforms continuous variables into ordinal variables and ensures the consistency of the classification results while effectively preserving the ordered nature of the data. Based on data transformation, this study applies the method designed by Luo et al. (29) to construct a causal Directed Acyclic Graph (DAG). Then, using the ordinal data causal analysis algorithm proposed by (30), ordinal causal effects (OCE) between ordered variables are calculated within the framework of the latent Gaussian DAG model. These findings offer valuable insights for optimizing rehabilitation strategies and provide the critical foundations for the development of effective prevention strategies. The rest of the article is structured as follows. In Section 2, we present a summary of previous approaches, including both Gaussian DAG models and the do-operator. In Section 3, we present our original contribution with the quantile threshold function and how to evaluate ordinal causal effects by combining the algorithm of OSEM and the Latent Causal Inference Framework. In Section 4, we use real-world data to illustrate the performance of causal effect estimation with latent DAG structures. Lastly, in Section 5, we discuss the potential prospects and limitations of the research and highlight possible directions for expanding the current work.

## 2 Background

### 2.1 Gaussian DAG-models

Probabilistic graphical models, which integrate graphical structures into probabilistic inference, are widely used and effective frameworks for studying these complex systems. The concept is to factorize the joint probability distribution *p* for the variables $\text{X}={\left(X_{1},\cdots ,X_{m}\right)}^{⊤}$ concerning a graph G=(V,E), where V is the set of vertices representing the variables and E the set of edges encoding the independence relationships (31, 32). Bayesian networks are a special class of probabilistic graphical models, where G is a directed acyclic graph (DAG) or named DAG models (33, 34). The joint probability distribution *p* can be specified by a set of parameters θ and factorizes based on G as:


(1) $$\begin{array}{l} p\left(\text{x}|\theta ,G\right)=p\left(x_{1},\ldots ,x_{m}|\theta ,G\right)=\overset{m}{\underset{i=1}{\prod }}p\left(x_{i}|{\text{x}}_{pa\left(i\right)},\theta_{i},G\right). \end{array}$$

Where $\theta =\cup_{i=1}^{m}\theta_{i}$, **x** is a realization of **X**, and we assume that the subsets ${\left\{\theta_{i}\right\}}_{i=1}^{m}$ are disjoint. Denote the parents of node *i* by *pa*(*i*)), where there is a directed edge from *j* to *i* if *j*∈*pa*(*i*). Thus, Equation (1) can also be interpreted as stating that a variable *x*_*i*_ is conditionally independent of its non-descendants, given its parents **X**_*pa*(*i*)_ in G. This is the Markov property (31).B=(G,θ) denotes a Bayesian network. Given a data sample X, learning a Bayesian network, therefore, involves estimating both the network structure G and θ. If the joint distribution of ***X*** is a Gaussian distribution, then


(2) $$\begin{array}{l} X\sim N\left(\mu ,\Sigma \right). \end{array}$$

In the case of Gaussian data, to address the uncertainty regarding the graphical structure, Maathuis et al. (35) provided lower bounds for causal effects after identifying a Markov equivalence class that is consistent with the data. By using a Bayesian approach, one can combine structure learning and effect estimation into a process that produces the posterior distribution of causal effects. A significant advantage is that this method accounts for both graphical and parameter uncertainty, as first proposed and demonstrated in a psychology application by Moffa et al. (36) for binary data.

where the matrix **Ω** = **Σ**^−1^ is symmetric, positive definite, and Markov relative to G. Under the assumption of normality, the Gaussian DAG model is almost always faithful to the DAG within the parameter space, which means that the conditional independence relationships implied by the distribution are precisely the same as those represented by the DAG through the Markov property (37). For a Gaussian DAG model, we can rewrite the factorization in Equation 1 as Equation (3): (ϕ denotes the normal univariate density function) (33).


(3) $$\begin{array}{l} p\left(\text{x}|G,\mu ,\Sigma \right)=\overset{m}{\underset{i=1}{\prod }}\phi \left(x_{i}|\mu_{i}\left({\text{x}}_{pa\left(i\right)}\right),\sigma_{i}^{2}\right). \end{array}$$

### 2.2 Do-operator

The do-operator is a fundamental concept in causal inference, proposed by Pearl (37). Provides a theoretical framework for quantifying the effects of interventions in a system. By modifying the observed probability distribution, the do-operator helps distinguish between correlation and causation. It is a core tool in causal inference and decision theory, allowing researchers to systematically answer the question “What would happen if?”

**Figure 1:**
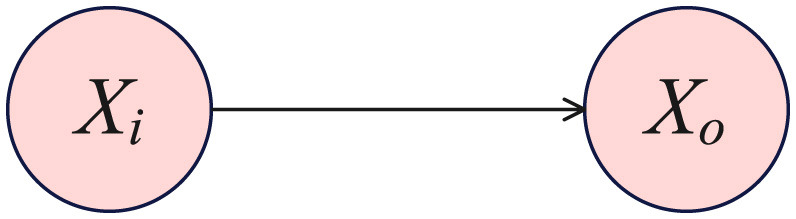
The outcome variable *X*_o_ resulting from a deterministic intervention on the intervention variable *X*_i_.

DAG provides an alternative approach to causal inference. Using DAG to describe the data generation process is appealing because the edges of the graph naturally represent the causal relationships between variables. Under a known DAG, the do-operator determines the causal effect of one variable on another. We have discussed the DAG model obtained by the Gaussian model, but we are interested in the causal relationships between the nodes, as shown in Figure 1.

Our goal is to determine the causal effect of variable *X*_i_ on the variable *X*_o_. We use Pearl's do-operator to describe the effects of intervention (37), where the distribution of *X*_o_ under an intervention in *X*_i_ is generally indicated as ℙ(*X*_o_ = *k*∣do(*X*_i_ = *l*)). Changes in the distribution or shifts in the distribution of the outcome variable across different levels of the intervention variable often serve as target estimands with practical significance (38). Evaluating and contrasting the change in the probability of *X*_o_ belonging to level *k*, when the intervention variable *X*_i_ is set to level *l*′ versus level *l* offers a measure of the distribution shift:


(4) $$\begin{array}{l} \mathbb{P}\left[X_{\text{o}}=k|\mathrm{do}\left(X_{\text{i}}=l^{\prime }\right)\right]-\mathbb{P}\left[X_{\text{o}}=k|\mathrm{do}\left(X_{\text{i}}=l\right)\right]. \end{array}$$

for each *l*≠*l*′ and $l,l^{\prime }\in \left\{1,\ldots ,L_{\text{i}}\right\}$ and *k*∈{1, …, *L*_o_}. We can evaluate ordinal causal effects (OCE) as represented by the target causal estimands in Equation (4).

## 3 Materials and methods

### 3.1 Quantile threshold function

In the data $\text{X}={\left(X_{1},\cdots ,X_{m}\right)}^{⊤}$, the random variable *X*_*k*_ = (*x*_1_, *x*_2_, ⋯ , *x*_*n*_), where *k* = 1, 2, ⋯ , *m*, often includes both continuous data and ordinal data. Before causal analysis, data must be organized and optimized to ensure precision and reliability of the results. Inconsistent data types can have a significant impact on analysis results. For example, in regression analysis, it is necessary to standardize the data to ensure consistent units of measurement. The objective of this paper is to analyze ordinal data, so the first step is to properly transform unordered data into ordinal data, which supports the subsequent analysis. Therefore, we propose a quantile threshold function (QTF) that transforms continuous variables into ordinal variables and ensures the consistency of the classification results while effectively preserving the ordered nature of the data.

In this paper, we apply the kernel density estimation to fit the probability density of the continuous variable *X*_*k*_ in Equation 5. A Gaussian kernel function is chosen as the smoothing kernel in Equation 6, which has the advantage of not requiring a predefined data distribution shape. Through bandwidth adjustment, it effectively approximates unknown distributions. This method overcomes the dependence on distributional assumptions inherent in traditional parametric methods.


(5) $$\begin{array}{l} \hat{f}\left(x\right)=\frac{1}{nh}\overset{n}{\underset{i=1}{\sum }}K\left(\frac{x-x_{i}}{h}\right). \end{array}$$

(6) $$\begin{array}{l} K\left(u\right)=\frac{1}{\sqrt{2\pi }}\exp\left(-\frac{1}{2}u^{2}\right). \end{array}$$

Where $\hat{f}\left(x\right)$ represents the probability density function, *K*(*u*) is the Gaussian kernel function, and *h* is the bandwidth, which is a key smoothing factor in kernel density estimation. We adopt the bandwidth selection method proposed by Ripley (39) in his book *Modern Applied Statistics with S* as the criterion (page 127) (39). The definition of the quantile threshold function is as follows:

Definition 1 (quantile threshold function). The random variable *X* = (*x*_1_, *x*_2_, ⋯ , *x*_*n*_) has a probability density function *f*(*x*) and a distribution function *F*(*x*). If there exists a non-negative real-valued function *g*(*x*) such that:


(7) $$g(x)=\{\begin{array}{ll} \text{0}, & \text{if }x\leq Q_{i},\\ \text{1}, & \text{if }Q_{i}<x\leq Q_{i+1},\\ \text{2}, & \text{if }x>Q_{i+1}, \end{array}.$$

where *Q*_*i*_ satisfies $F\left(Q_{i}\right)=\overset{Q_{i}}{\underset{-\infty }{\int }}f\left(x\right)dx=\frac{i}{4},i\in \left\{1,2\right\}$, then, the function *g*(*x*) is called quantile threshold function

*g*(*x*) is also a random variable. The value of *x* ≤ *Q*_*i*_ in *X* is defined as the lower level (assigned a value of 0), and within the *Q*_*i*_<*x* ≤ *Q*_*i*+1_ interval is defined as a medium level (assigned a value of 1), and we classify values of *x*>*Q*_*i*+1_ as a high level (assigned a value of 2). Based on the above definition, we can naturally deduce the following conclusion.

Proposition 1. The sum of the probabilities of the three categories equals 1, that is, $\overset{2}{\underset{j=0}{\sum }}\mathbb{P}\left[g\left(x\right)=j\right]=1$

Proof. See the Supplementary material: Proof of Proposition 1.

Definition 1 extends to multiple classification scenarios, as shown in the following Definition 2.

Definition 2. The random variable *X* = (*x*_1_, *x*_2_, ⋯ , *x*_*n*_) has a probability density function *f*(*x*) and a distribution function *F*(*x*). If there exists a non-negative real-valued function *g*(*x*) such that:


(8) $$g(x)=\{\begin{array}{ll} \text{0}, & \text{if }x\leq Q_{i},\\ \text{1}, & \text{if }Q_{i}<x\leq Q_{i+1},\\ \text{2}, & \text{if }Q_{i+1}<x\leq Q_{i+2},\\ \vdots , & \vdots ,\\ n, & \text{if }x>Q_{i+n-1}, \end{array}.$$

where *Q*_*i*_ satisfies $F\left(Q_{i}\right)=\overset{Q_{i}}{\underset{-\infty }{\int }}f\left(x\right)dx=\frac{i}{n+2},i\in \left\{1,2,\cdots ,n\right\}$.

Proposition 2. The sum of the probabilities of the categories *n*+1 is equal to 1, that is, $\overset{n}{\underset{j=0}{\sum }}\mathbb{P}\left[g\left(x\right)=j\right]=1$.

Proof. See the Supplementary material: Proof of Proposition 2.

Proposition 3. $\underset{n\to \infty }{\lim}\mathbb{P}\left[g\left(x\right)=n\right]=0$.

Proof. See the Supplementary material: Proof of Proposition 3.

From Proposition 3, we know that when performing ordered classification on random variables, the classification must be finite. Therefore, the function *g*(*x*) achieves a smooth transition from continuous variables to ordinal variables. *g*(*x*) provides a structured data representation that maintains both information retention and interpretability for subsequent analysis.

In this paper, we use *n* = *i* = 2 as the classification criterion. After the classification is completed, we conduct hypothesis testing on the results. This study employs the Analysis of Variance (ANOVA) method to verify the significance of differences between groups for reconstructed ordinal variables (40). Specifically, our objective is to determine whether the differences among the three groups are significant, which can be achieved by testing whether the means of each group are the same. We set the significance level at α = 0.05. Let


$$H_{0}:\mu_{0}=\mu_{1}=\mu_{2}$$

$$H_{1}:\exists \;i,j\in \left\{0,1,2\right\}\;\text{s.t. }\mu_{i}\neq \mu_{j}$$

Where μ_0_, μ_1_, and μ_2_ are the means of the three groups. If *p* < 0.05, then *H*_1_ holds, indicating that there are significant differences among the three groups, which suggests that the above classification is effective. Conversely, if *H*_0_ holds, it indicates that the classification levels are not significant.

### 3.2 Latent Gaussian DAG model

We obtained ordinal data $X^{*}={\left(X_{1},\cdots ,X_{m}\right)}^{⊤}$ by the function *g*(*x*). What we are interested in is the DAG that represents the relationships between ordinal variables. We introduce the Gaussian DAG-models in Subsection 2.1, but this model is built under the assumption that the data follow a Gaussian distribution. Therefore, for the construction of the DAG model for ordered data, we have used the OSEM algorithm proposed by Luo et al. (29). By assuming that each ordinal variable is obtained by marginally discretising a set of Gaussian variables, we can get the ordinality amongst the categories. And Gaussian variables jointly follow a DAG structure G=(V,E). The OSEM algorithm provides a new framework that effectively learns Bayesian networks from ordinal data and captures the orderliness among categories.

Let X^*^ be a set of *m* ordinal variables, where *X*_*k*_ takes values in the collection of {τ(*k*, 1), τ(*k*, 2), …, τ(*k, L*_*k*_)} and τ(*k*, 1) <τ(*k*, 2) <⋯ <τ(*k, L*_*k*_), *k* = 1, …, *m*. We assume that the number of levels *L*_*k*_≥2, therefore, each variable should at least be binary. It is typical to set τ(*k, l*) = *l*−1 for all 1 ≤ *l* ≤ *L*_*k*_, i.e. τ(*k*, 1) = 0, τ(*k*, 2) = 1, and so on. Further, we assume that each *X*_*k*_ is obtained by discretising an underlying Gaussian variable *Y*_*k*_ using the thresholds −∞ =:α(*k*, 0) <α(*k*, 1) <⋯ <α(*k, L*_*k*_−1) <α(*k, L*_*k*_): = ∞. Let $\alpha_{i}={\left(\alpha \left(k,0\right),\ldots ,\alpha \left(k,L_{k}\right)\right)}^{⊤}$ and $\alpha ={\left\{\alpha_{k}\right\}}_{k=1}^{m}$. Thus *X*_*k*_ is defined by the following rule:


(9) $$X_{k}=\left\{ \begin{array}{l} \tau (k,1)\text{ if }Y_{k}\in (-\infty ,\alpha (k,1))\\ \;\vdots \\ \tau \left(k,L_{k}\right)\text{ if }Y_{k}\in \left[\alpha \left(k,L_{k}-1\right),+\infty \right) \end{array}\right.$$

**Figure 2:**
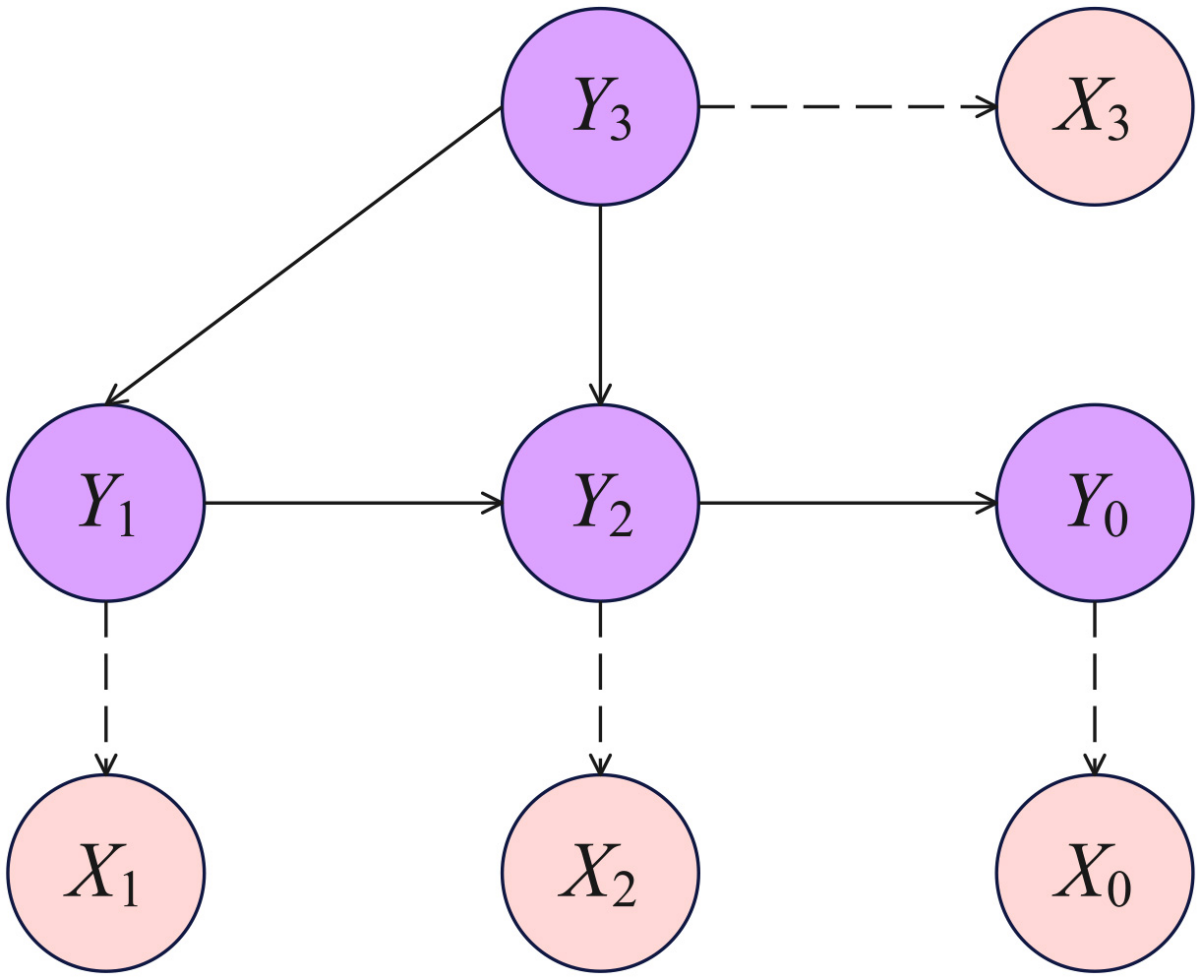
Example of latent Gaussian four-nodes DAG. Variables *X*_*k*_, *k* = 0, …3 are ordinal, each obtained by discretising a latent variable *Y*_*k*_ with associated Gaussian parameters θ_*k*_. Ordinal nodes are dashed for clarity.

Of course, $\text{Y}={\left(Y_{1},\cdots ,Y_{m}\right)}^{⊤}$ are unobservable, and we observe *X*_*k*_ obtained from the continuous variables by discretisation. The diagram in Figure 2 offers a visual depiction of the setup in an example case with a few variables.

Formally, Luo et al. (29) proposed a DAG model for ordinal variables based on latent Gaussian variables. The model makes the following optimization definition based on Equation 2. The detailed derivation process can be found in the paper (29).


(10) $$\begin{array}{l} Y_{k}|y_{\text{pa}(k)},\vartheta_{k},G\sim N\left(\mu_{k}+\sum_{j\in \text{pa}(k)}b_{jk}\left(y_{j}-\mu_{j}\right),v_{k}\right),\\ \mathbb{P}\left(X_{k}=\tau (k,l)|Y_{k}=y_{k},\alpha_{k}\right)=⊮\left(y_{k}\in [\alpha (k,l-1),\alpha (k,l)]\right),\\ l=1,\ldots ,L_{k},\\ p(x,y|\theta ,G)=\prod_{k=1}^{n}\phi \left(y_{k}|y_{\text{pa}(k)},\vartheta_{k},G\right)p\left(x_{k}|y_{k},\alpha_{k}\right), \end{array}$$

The OSEM combines the multinomial probit model (41) and the structural EM algorithm of (42) to solve the problem of learning Bayesian networks from ordinal data. Specifically, the method proposes an iterative scoring and search strategy - the Ordinal Structural EM (OSEM) algorithm for learning Bayesian networks from ordinal data.

### 3.3 Causal effects in the latent Gaussian DAG model

Consider the general latent Gaussian DAG-model of Section 3.2. We are interested in computing the target causal estimand in Equation 4. For example, in Figure 2, the intervention variable *X*_*i*_ is *X*_1_, and the outcome variable *X*_*o*_ is *X*_0_. The Equation 4 can be written as Equation 11, representing the OCE on *X*_*o*_ of an intervention on *X*_*i*_. When the intervention variable *X*_i_ is set to level *l*′ versus level *l* offers a measure of the distribution shift (37):


(11) $$\begin{array}{l} \mathbb{P}\left[X_{\text{o}}=\tau (\text{o},k)|\mathrm{do}\left(X_{\text{i}}=\tau \left(\text{i},l^{\prime }\right)\right)\right]-\mathbb{P}[X_{\text{o}}=\tau (\text{o},k)\\ \;|\mathrm{do}\left(X_{\text{ i}}=\tau (\text{i},l)\right)] \end{array}$$

The direct computation of Equation 11 would result in 0 for each level of the intervention and outcome variables because there is no causal path between the ordinal *X*_*i*_ and *X*_*o*_ in the DAG. But it is evident that they are causally related to each other by *Y*. Therefore, we can consider that if we intervene on the latent variable *Y*_i_ in a way that changes the level of its ordinal child variable *X*_i_, and then compute the effect of this intervention on the latent parent *Y*_o_ of *X*_o_, it is possible that the level of *X*_o_ could also change as a result. The potential change in the level of *X*_o_ resulting from an intervention on the latent parent of *X*_i_ is the OCE studied in article (30). Using the $\alpha ={\left\{\alpha_{k}\right\}}_{k=1}^{m}$, the target causal estimand in Equation 11 on the ordinal variables can be equivalently computed as the following [(30); Definition 1,page9]:


(12) $$\begin{array}{l} {\text{OCE}}_{\text{io}}\left(k,l\to l^{\prime }\right)=\mathbb{P}[Y_{\text{o}}\in [\alpha (\text{o},k-1),\alpha (\text{o},k)]\\ \;|\mathrm{do}\left(Y_{\text{i}}\in \left[\alpha \left(\text{i},l^{\prime }-1\right),\alpha \left(\text{i},l^{\prime }\right)\right]\right)]\\ \;-\mathbb{P}[Y_{\text{o}}\in [\alpha (\text{o},k-1),\alpha (\text{o},k)]\\ \;|\mathrm{do}\left(Y_{\text{i}}\in [\alpha (\text{i},l-1),\alpha (\text{i},l)]\right)]. \end{array}$$

for each $1\leq k\leq L_{\text{o}},1\leq l,l^{\prime }\leq L_{\text{i}}$, with *l*≠*l*′. The definition of OCE is anti-symmetric for the initial and end level of the intervention variable, implying that


(13) $$\begin{array}{l} {\text{OCE}}_{\text{io}}\left(k,l\to l^{\prime }\right)=-{\text{OCE}}_{\text{io}}\left(k,l^{\prime }\to l\right). \end{array}$$

**Figure 3:**
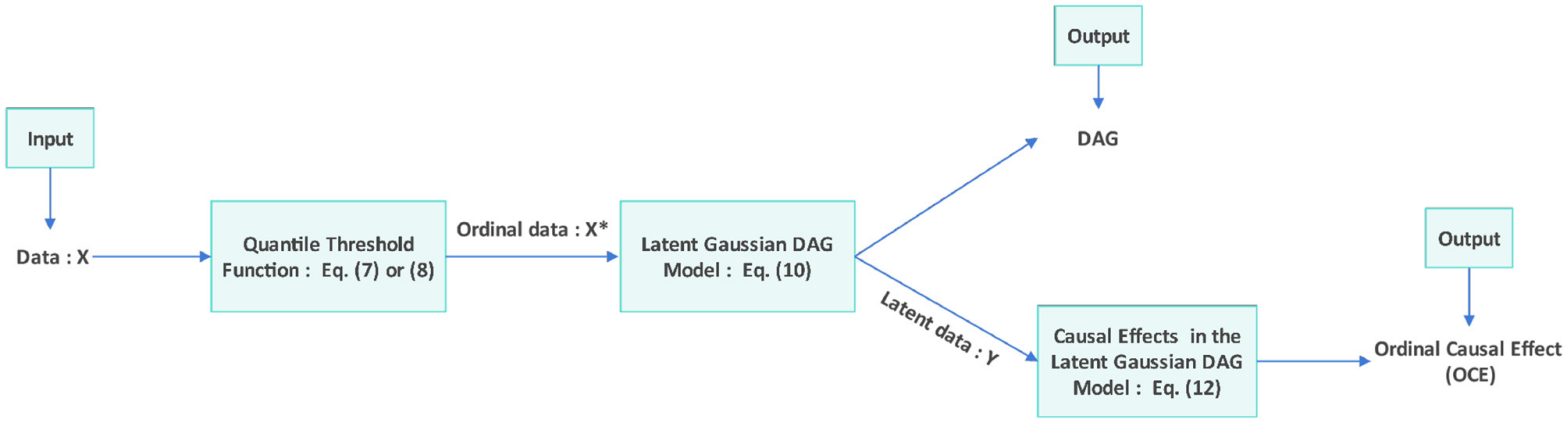
Flowchart of the algorithm: after inputting the initial data **X**, the QTF is applied to obtain the ordinal data X^*^. The Latent Gaussian DAG Model (29) identifies the latent data **Y** and the DAG. Then, we compute the causal effects in the Latent Gaussian DAG Model (30) to obtain the ordinal causal effect.

Based on the above Equation 12, the Latent Gaussian DAG-model establishes a relationship for calculating the intervention effect between ordinal variables. This allows for the computation of the OCE between variables X^*^, which is equivalent to the intervention effect between variables *Y*. The (30) provides a detailed proof and derivation of Equation 12 in both the main text and the Appendix. Building on this result, Scauda et al. (30) proposed Proposition 5 - a method for computing OCE. The specific details can be found in Proposition 5 (Computation of the Ordinal Causal Effect) on page 12 of the (30). According to Proposition 5, we can calculate the OCE efficiently. Figure 3 illustrates the flowchart of the proposed algorithm.

## 4 Results

### 4.1 Data

Maintaining an injury-free condition is a crucial factor for success in sports. Although injuries are difficult to predict, the application of emerging technologies and data science can offer valuable insights. Even with a well-specified model, inaccurate data can compromise causal analysis. Data quality is often more critical than sample size (43), particularly in sports science, where physiological measures (e.g., maximal oxygen uptake, gene transcription activity) are inherently noisy due to biological and technical variability. Additionally, exercise intervention studies face challenges such as participant dropout, missing data, measurement errors, and inconsistencies in data processing, all of which hinder reliable causal interpretation (44).

This study utilizes a comprehensive training log dataset from Kaggle (45, 46) collected by a Dutch team in 2012–2019. This dataset, developed by Lövdal et al., employs machine learning to predict injuries based on data that focuses on middle- and long-distance events (800m to the marathon), with detailed performance records for 74 athletes (27 females and 47 males). The dataset adhered to the ethical principles of the *Declaration of Helsinki* and received formal approval from the ethics committee (45). This study follows the data structure established in (45), utilizing the Day approach [(45), page 1523, Table 1], with this research focusing exclusively on the data from a single day to analyze the causal effects. This dataset contains a total of 42,766 samples. Table 1 presents detailed characteristics of the variables. Variables 1, 6, and 11 are directly considered ordinal, while the remaining variables require ordinal classification using QTF.

**Table 1:** List of Variables and Descriptions.

**ID**	**Variable**	**Description**
1	Sessions	Number of trainings completed
2	Totalkm	Number of kilometers covered by running
3	Kmmidlle	Number of kilometers covered in intensity zones 3 and 4
4	Kmhigh	Number of kilometers covered in intensity zone 5
5	Kmsprinting	Number of kilometers covered with sprints
6	Strengthtraining	Whether the day included a strength training session
7	PerceivedtrainingSuccess	Athlete's self-rating of how well the session went.
8	Hoursalternative	Number of hours spent on cross training
9	Perceivedexertion	Athlete's self-rating of fatigue after the session.
10	Perceivedrecovery	Athlete's self-rating of restfulness before the session.
11	Injury	Whether injured

Dataset from Kaggle: Lovdal et al. (45), Injury Prediction Dataset (45).

### 4.2 Analysis

We simulated the probability density function for continuous variables and classified the variables based on QTF. The graph of the probability density function and its corresponding quantile ranges are presented in the Supplementary Figure 1. Taking variable 2 as an example, from Supplementary Figure 1 we can observe that *Q*_2_ and *Q*_3_ divide the range of the values of the variable into three parts. The portion less than *Q*_2_ is defined as low level and assigned a value of 0. The values between *Q*_2_ and *Q*_3_ are categorized as medium level and assigned a value of 1. Finally, values greater than *Q*_3_ are defined as high level and assigned a value of 2. From Supplementary Figure 1, we can see that the probability of values at the low level (green) is 0.5, the probability at the medium level (orange) is 0.25, and the probability at the high level (purple) is also 0.25. Through this method, we transform the variable from a continuous variable to an ordinal variable. Subsequently, we perform ANOVA on the three groups levels and conduct a significance test (Table 2) (40).

**Table 2:** ANOVA for the three group levels and significance testing based on the QTF.

	**Group (mean** ±**SD)**_*****n*****_			**F**	**p**
**Ordinal**	**“0”**	**“1”**	**“2”**		
Variable 2	3.186 ± 3.991_30437_	12.882 ± 0.98_5996_	20.016 ± 5.2547_6333_	55364	0.001^⋆⋆⋆^
Variable 3	0.269 ± 1.088_40762_	7.281 ± 0.621_1008_	11.298 ± 4.244_996_	52351	0.001^⋆⋆⋆^
Variable 4	0.178 ± 0.697_40071_	5.299 ± 0.611_1726_	8.781 ± 3.237_969_	77658	0^⋆⋆⋆^
Variable 5	0.017 ± 0.084_41315_	0.899 ± 0.113_764_	2.536 ± 2.666_687_	19913	0.001^⋆⋆⋆^
Variable 7	0.087 ± 0.190_26234_	0.689 ± 0.050_8540_	0.850 ± 0.063_7992_	102575	0.001^⋆⋆⋆^
Variable 8	0.050 ± 0.020_40181_	1.345 ± 0.158_1269_	2.514 ± 1.155_1316_	61170	0^⋆⋆⋆^
Variable 9	0.047 ± 0.067_22291_	0.293 ± 0.083_10255_	0.639 ± 0.129_10220_	156092	0^⋆⋆⋆^
Variable 10	0.055 ± 0.074_22844_	0.234 ± 0.043_9811_	0.477 ± 0.117_10111_	96383	0^⋆⋆⋆^

^⋆⋆⋆^*p* < 0.001; The three groups of data (“0”, “1”, “2”) are presented as mean ± standard deviation (Mean ± SD)_*n*_, with *n* representing the sample size, and the *F*, *p* values are also listed.

The mean values of the variable increase monotonically from group “0” to group “2”, and the standard deviations also progressively increase. For variable 2, the means and standard deviations at different levels are as follows: In group “0”, the mean is 3.186, the standard deviation is 3.991, and the sample size is 30, 437; in group “1”, the mean is 12.882, the standard deviation is 0.98, and the sample size is 5, 996; in group “2”, the mean is 20.016, the standard deviation is 5.2547, and the sample size is 6, 333. Other variables exhibit similar patterns, where the mean in group “0” is relatively small, while the means in groups “1” and “2” are significantly higher (e.g., for variable 5, the mean increases from 0.017 to 2.536). Several variables have extremely high F-values (e.g., 156, 092 for the variable), showing that the between-group variance is far greater than the within-group variance. The ANOVA results indicate that the differences in means across groups are statistically significant (*p* < 0.05), suggesting that the distribution of the variable differs significantly across levels and shows a clear gradient pattern (group “0” <“1” <“2”). This result implies that the method used can effectively distinguish differences between levels, leading to the rejection of the null hypothesis *H*_0_ and the acceptance of the alternative hypothesis *H*_1_.

In the Supplementary material, we present the ANOVA results for the three discretization methods: equal-width, equal-frequency, and k-means. Supplementary Table 1 shows the ANOVA results for equal-width. This method uses intervals of the same width, making it simple and intuitive. However, the ANOVA results indicate that its F-value is lower, suggesting the between-group differences are less significant than with the QTF method. Moreover, for outlier variables (such as Variable 4 or Variable 5), equal-width fails to reflect the data distribution accurately. Supplementary Table 2 shows the ANOVA results for equal-frequency. This method ensures that each interval contains roughly the same number of samples. When sample values are highly concentrated and repeated, the resulting zero mean and standard deviation for some groups reduces the interpretability of the data. Supplementary Table 3 presents the results of k-means clustering. The ANOVA shows large F-values for the three clusters, suggesting significant between-group differences. However, k-means is a typical data-driven method with unfixed boundaries. If clusters are ordered by their mean values to define “low,” “medium,” and “high” levels, the boundaries will change with the data because cluster centers are randomly initialized. This randomness weakens theoretical interpretability.

**Figure 4:**
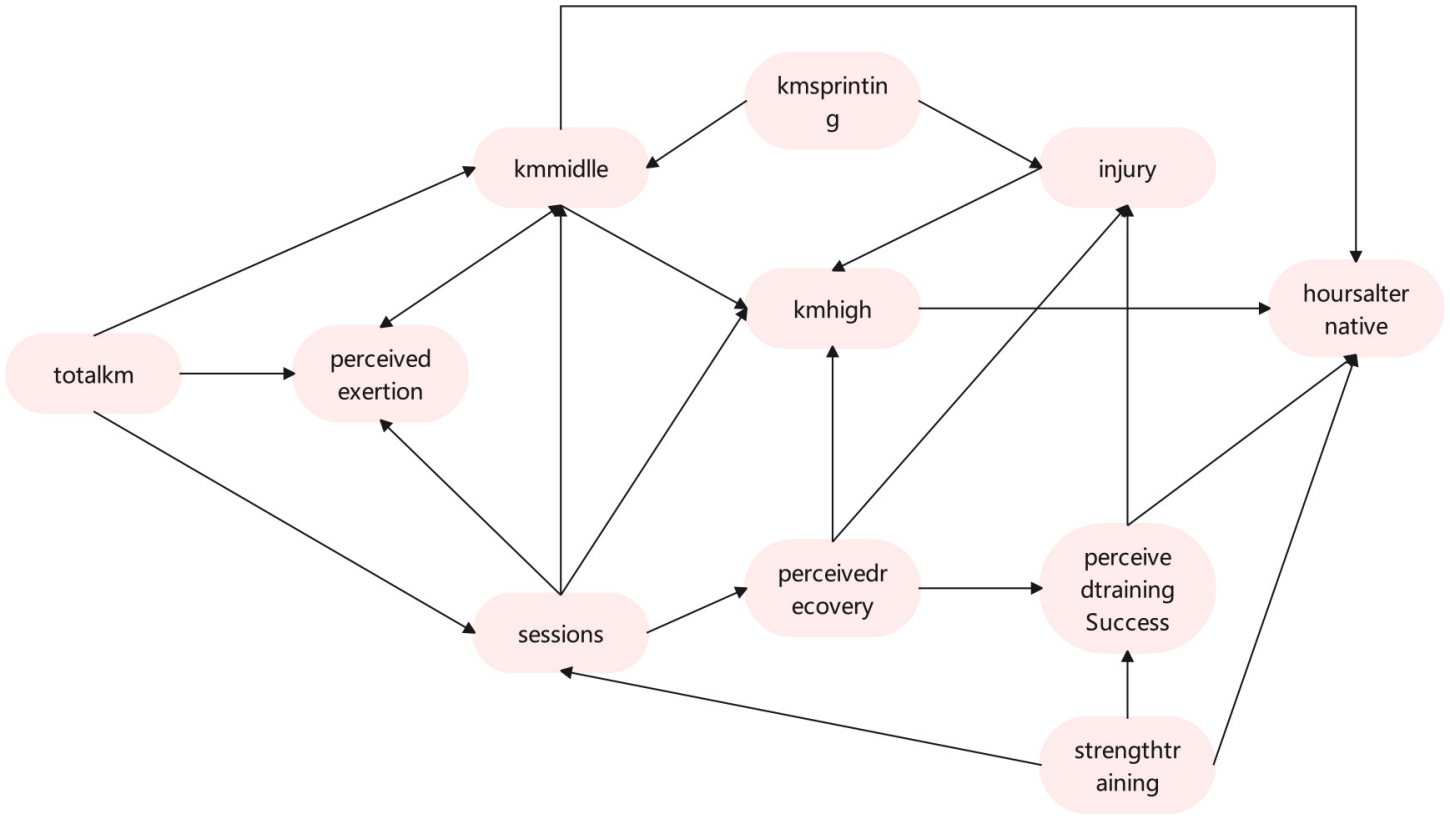
CPDAG of the hidden network structure estimated via the OSEM algorithm (29) for the ordinal data of Section 4.1.

We followed the approach described in Luo et al. to derive DAG estimates from 500 bootstrap samples of the data (29, 30), utilizing the OSEM algorithm with a Monte Carlo sample size of *K* = 5 and a penalty coefficient of λ = 6. And the resulting CPDAG is shown in Figure 4.

Figure 4 illustrates the causal relationships between different variables, highlighting both direct and indirect variables surrounding the **Injury** variable. The direct influencing variable for **Injury** is **Kmsprinting**, which has a significant impact on the occurrence of **Injury**. **Kmsprinting** training typically places high stress on muscles, joints, and ligaments, especially when the intensity is excessive or recovery is insufficient, making it prone to cause tissue damage or excessive fatigue. **Kmsprinting** may lead to rapid muscle contraction and high load in a short period, thereby increasing the risk of exercise-related injuries. **Perceivedrecovery** and **Perceivedtrainingsuccess** are also direct variables influencing **Injury**. **Perceivedrecovery** plays a critical role in injury risk. When perceived recovery is poor, muscles and joints may not withstand higher loads, potentially leading to improper movement and decreased endurance, thus increasing the risk of injury. **Perceivedtrainingsuccess** directly affects injury risk. If an athlete perceives training success as high, it may indicate good physical condition and effective recovery, thereby reducing the risk of injury. Conversely, a lower perception of training success may indirectly reflect accumulated fatigue and insufficient recovery, increasing the probability of injury.

**Injury**'s indirect influencing variables, Pathway 1 is **Totalkm**→**Sessions**→**Perceivedrecovery**→**Injury**: Increasing total running distance can lead to higher training intensity and frequency. A rise in total distance is often accompanied by increased training frequency, which may result in insufficient recovery time. Excessive training frequency and load can compromise recovery quality, leading to poor perceived recovery and indirectly increasing the risk of injury. If increases in running distance and frequency are not balanced with adequate recovery and proper load management, the perceived recovery level may decline, significantly elevating the risk of injury.

Pathway 2 is **Strengthtraining**→**Perceivedtrainingsuccess**→**Injury**: **Strengthtraining** enhances muscle strength, joint stability, and exercise efficiency, thereby improving athletic performance and training outcomes. It also increases athletes confidence and perception of training success, which may indirectly indicate improved physical adaptation and recovery levels. **Perceivedtrainingsuccess** can reduce the risk of injury, as the body is in better physical condition, movements are more precise, and energy distribution is more efficient. Conversely, a lower perception of training success may have the opposite effect. Based on the above analysis, it is recommended to control the intensity and frequency of **Strengthtraining** to prevent muscle injuries caused by overtraining. Attention should be placed on athletes **Perceivedrecovery** and sense of **Perceivedtrainingsuccess**, and by adjusting the training plan, recovery outcomes can be effectively improved.

**Figure 5:**
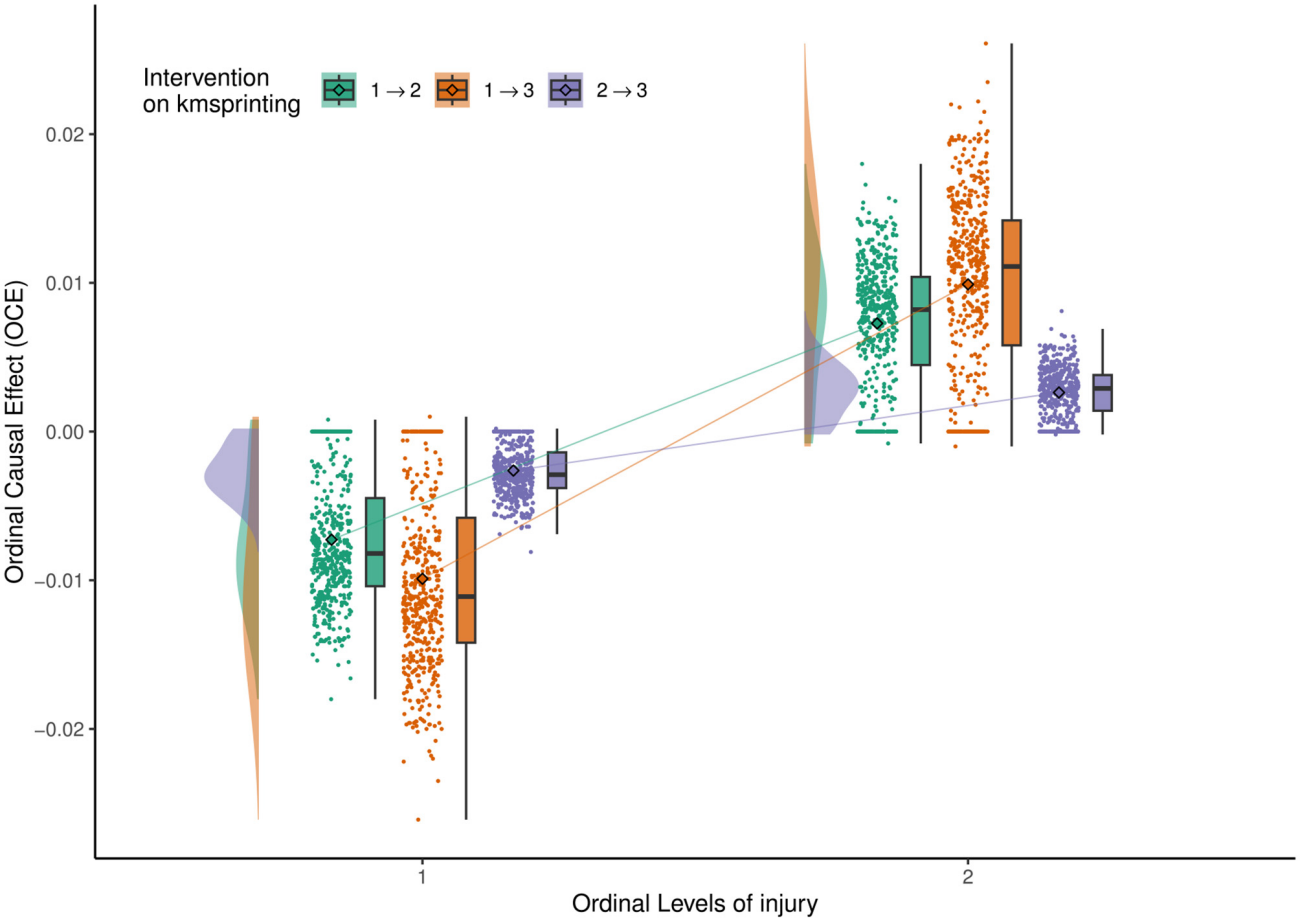
Ordinal Causal Effect of **Kmsprinting** (variable 5) on **Injury** (variable 11). The solid line connects the means of the OCEs (represented by diamond points) across different levels of the outcome variable, for each possible shift of the intervention variable.

To visually represent the bootstrapped estimates, we present the adjacency matrices of the DAG derived using OSEM, converted to CPDAG, as a heatmap in Supplementary Figure 2 of the Supplementary material. The intensity of each cell corresponds to the frequency with which each edge appears in the bootstrapped samples. The shade in the grid indicates the proportion of times a directed edge occurs in the 500 bootstrapped CPDAG, with an undirected edge being split equally between both directions. Darker shading corresponds to a higher frequency of the respective directed edge. Additionally, we examine the causal relationship along the most frequently observed directed edge in the 500 bootstrapped CPDAG by estimating the ordinal causal direct effects of **Kmsprinting** (variable 5) on **Injury** (variable 11) within the sample's DAG. Raincloud plots, which incorporate histograms and boxplots of the estimated effects, are displayed in Figure 5.

Figure 5 shows the ordinal causal direct effects of **Kmsprinting** (variable 5) on **Injury** (variable 11). “**Injury=1**” indicates no injury, while the level of “**Injury=2**” indicates injury. We use the do-operator to describe this result.

When the “**Injury=1**”, and the level of variable 5 changes from 1 → 2, the OCE <0. That means ℙ[injury = 1∣*do*(kmsprinting = 2)]−ℙ[injury = 1∣*do*(kmsprinting = 1)] <0. This result suggests that increasing the intensity of sprint training significantly increases the injury risk for uninjured athletes. High-intensity sprint training may lead to excessive load on muscles and joints, exceeding the body's ability to adapt, thereby increasing the likelihood of injury. Therefore, for uninjured athletes, maintaining a lower level of sprint training helps to minimize the injury risk. If the sprint training level changes from 1 → 3, or from 2 → 3, the OCE value remains negative. This finding indicates that increasing the intensity of sprint training has a significant impact on the injury probability for uninjured athletes, especially during the transitions from 1 → 2 and from 1 → 3. Among these three intervention levels, the absolute value of the OCE mean is largest for the intervention from 1 → 3, suggesting that transitioning directly from low to high intensity training is particularly dangerous for uninjured athletes. Therefore, training plans need to be carefully designed.

When the “**Injury=2**”, and the level of variable 5 changes from 1 → 2, the OCE > 0. That means ℙ[injury = 2∣*do*(kmsprinting = 2)]−ℙ[injury = 2∣*do*(kmsprinting = 1)]>0. When the sprint training level increases from 1 → 2 or 1 → 3, the probability of injury significantly increases. For injured athletes, continuing to increase the sprint training intensity before full recovery can worsen the existing injury or lead to incomplete recovery, which significantly increases the risk of injury. During this stage, athletes should avoid increasing sprint training intensity and prioritize basic training and recovery. When the sprint training level changes from 2 → 3, the OCE for both injured groups is more concentrated, with the mean close to 0, indicating that the impact of increasing from medium to high-intensity sprint training on injury is relatively small. This limited effect may be due to the athletes' adaptation to medium-intensity training and their more stable physical condition. Therefore, after sufficient medium-intensity training, gradually increasing high-intensity sprint training does not significantly increase the injury risk.

In summary, the transition from low to medium intensity (1 → 2) is a critical period for injury risk, requiring close monitoring of an athlete's physical condition and recovery. After reaching medium intensity, transitioning to high intensity is relatively safe, as the body has developed some level of adaptation, resulting in a lower injury risk. For uninjured athletes, it is recommended to prioritize a progressive increase in training load and avoid a direct jump from low to high intensity (1 → 3). For injured athletes, high-intensity sprint training should be strictly limited during the recovery period, and priority should be given to recovery training. High-intensity sprint training requires a focus on recovery quality after training to ensure athletes maintain a good recovery state during the gradual increase in intensity, minimizing fatigue accumulation and injury risk. The **Kmsprinting** has a significant causal effect on **Injury**. The risk from medium to high intensity (2 → 3) is relatively small, suggesting that training upgrades should be based on the athlete's adaptation. Scientifically planning sprint training intensity and pace, combined with recovery training, helps reduce injury probability while improving training effectiveness and safety.

**Figure 6:**
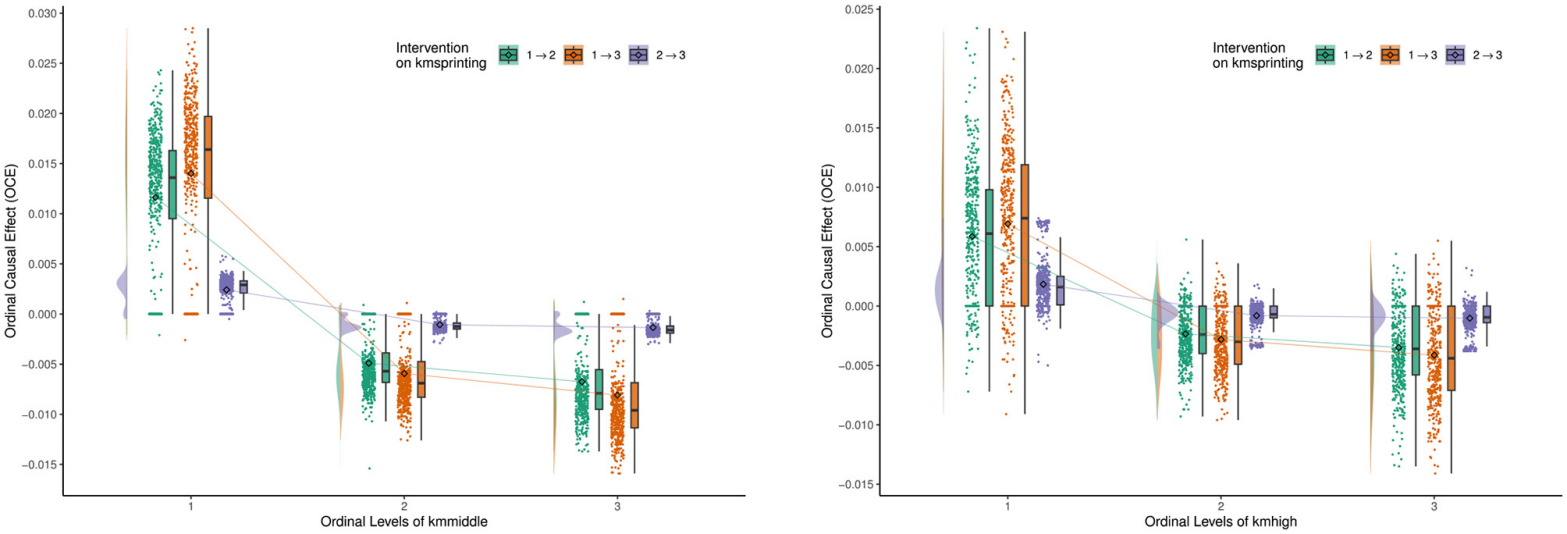
Ordinal causal effect of **Kmsprinting** (variable 5) on **Kmmiddle** (variable 3) and **Kmhigh** (variable 4).

Figure 6 shows the ordinal causal direct effects of **Kmsprinting** (variable 5) on **Kmmiddle** (variable 3) and **Kmhigh** (variable 4). The change of variable 5 from low level to medium level (1 → 2) shows that for low-level **Kmmiddle** athletes, an increase in sprinting results in a positive OCE value, indicating that improvements in sprinting may indirectly help these athletes enhance their middle-distance running ability. However, for medium and high-level **Kmmiddle** athletes, the OCE value is negative, suggesting that an increase in sprinting may decrease their performance in middle-distance running. A similar pattern applies to **Kmhigh** athletes. For low-level **Kmhigh** athletes, an increase in sprinting may improve their high-intensity running ability. In contrast, for medium and high-level athletes, an increase in sprinting may limit their high-intensity running ability.

It is worth noting that the OCE for **Kmhigh** is more scattered, indicating significant variation in the impact of sprinting on high-intensity running. The change of variable 5 from medium level to high level (2 → 3) shows that for both **Kmmiddle** and **Kmhigh**, the OCE are close to 0. This result suggests that once athletes have adapted to a certain level of sprinting load, further increases in sprint intensity have minimal effect on middle-distance running and high-intensity running performance. For athletes with weaker middle-distance and high-intensity running abilities, increasing sprint training can indirectly enhance their running abilities by improving neuromuscular adaptation and rapid power output. For athletes who already possess strong middle-distance and high-intensity running capabilities, increasing sprint training may lead to overloading or accumulation of fatigue, thereby affecting other running abilities. Once sprinting has reached a high level, athletes' bodies gradually adapt to the high-intensity load, and further increases in sprinting have limited intervention effects on other running abilities.

Therefore, we can conclude that for low-level athletes, appropriately increasing sprint training can help improve their middle-distance and high-intensity running abilities. For medium and high-level athletes, the ratio of sprinting to other running training must be balanced to avoid excessive sprint loads that may affect overall running performance. Regularly monitoring athletes' performance in different running abilities and adjusting the sprinting load based on data feedback will ensure maximized training effects while preventing fatigue accumulation. As sprint load increases from medium to high intensity (2 → 3), gradual adaptation should be emphasized to avoid sudden increases in training intensity that could negatively impact middle-distance or high-intensity running abilities. Through reasonable design and adjustment of sprint training, coaches and athletes can enhance specific abilities while minimizing the risk of injuries caused by overtraining, ultimately achieving comprehensive optimization of athletic performance.

**Figure 7:**
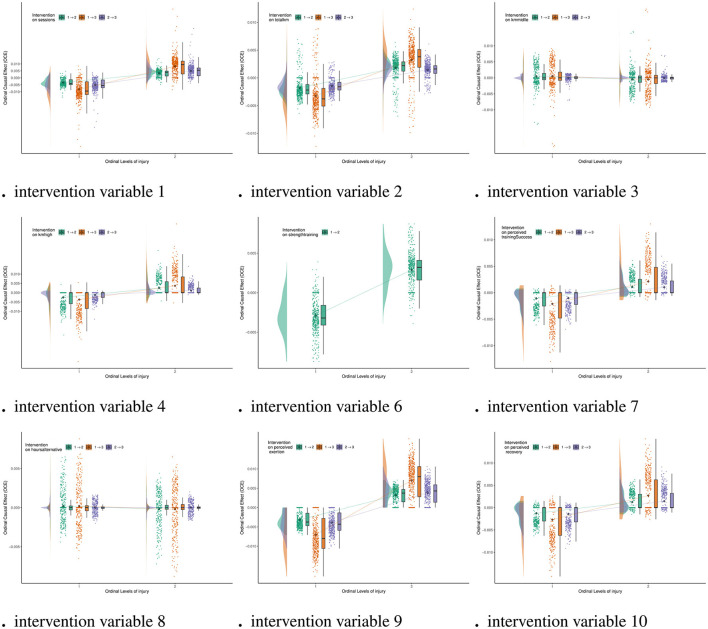
Ordinal causal effect of variable on **Injury** (variable 11).

Figure 7 shows the ordinal causal direct effects of other variables on **Injury** (variable 11). From Figure 7, we can observe that the OCE of **Kmmiddle**(variable 3) and **Hoursalternative**(variable 8) on injury are close to zero across various levels of change. This finding indicates that these two variables have a minimal impact on injury risk. **Kmmiddle** typically involves moderate-intensity training, and athletes' bodies are generally more adaptable, so it does not significantly increase or decrease injury risk. **Hoursalternative** mainly refers to low-intensity recovery exercises, which place minimal stress on the body, thus limiting their impact on injury risk. These two variables can be part of a stable training load to alleviate the physical stress from high-intensity training, thereby improving the overall safety of the training program.

In contrast, the changes in **Strengthtraining**(variable 6) show the most significant fluctuations in the OCE for injury, indicating a significant causal intervention effect. This finding suggests that strength training can enhance athletic performance but may also increase injury risk if the intensity is too high or the progression is too rapid. High-intensity strength training places a heavy load on muscles and joints, and without adequate recovery or adaptive training, it can lead to muscle strains, ligament injuries, and other issues. Additionally, **Strengthtraining**'s effects vary significantly among individuals, as different athletes have differing capacities to tolerate intensity and recover. This further amplifies its impact on injury risk. Therefore, strength training programs must strictly control intensity and progression, prioritizing the development of foundational strength and movement stability. This approach helps mitigate injury risk caused by excessive or improperly planned training.

The Pathway 2 analysis of the CPDAG in Figure 4 shows that **Strengthtraining**(variable 6) indirectly influences injury risk through Perceived **Perceivedtrainingsuccess**(variable 7), which supports the conclusion that their impact on injury risk is relatively limited. In practical sports, this result suggests that strength training should pay particular attention to intensity management. Proper planning of load progression is key to avoiding sports injuries, especially during high-intensity strength training, where it is important to incorporate restorative training [such as extending **Hoursalternative**(variable 8) ]. **kmmiddle**(variable 3) and **Hoursalternative**(variable 8) can serve as transition phases for high-intensity training, helping athletes gradually adapt to higher loads and reduce fatigue accumulation. Combining feedback from **Perceivedrecovery**(variable 10) and **Perceivedtrainingsuccess**(variable 7) allows for the real-time optimization of training plans, ensuring a balance between performance enhancement and injury risk. Through careful design and dynamic adjustment of training plans, athletes can not only reduce the risk of training-induced injuries but also improve overall training quality. Additionally, we provide the causal direct effects of other variables in the Supplementary Figure 3 for further reference.

## 5 Discussion

In this work, we propose a quantile threshold function (QTF) that transforms continuous variables into ordinal variables, and ensures the consistency of the classification results while effectively preserving the ordered nature of the data. Based on data transformation, this study applies the method designed by Luo et al. (29) to construct a causal Directed Acyclic Graph (DAG). Then, using the ordinal data causal analysis algorithm proposed by Scauda et al. (30), the ordinal causal effects (OCE) between ordered variables are calculated within the framework of the latent Gaussian DAG model. These findings offer valuable insights for optimizing rehabilitation strategies and provide the critical foundations for the development of effective prevention strategies. The use of causal diagrams will facilitate the organization of key concepts and ideas related to athletic injury causation within a well-defined causal framework. This approach enables the exploration of specific causal links and underlying assumptions through appropriate scientific methods. The findings not only advance methodological understanding but also provide a robust theoretical foundation for optimizing rehabilitation protocols and designing effective injury prevention strategies, ultimately contributing to a more precise and thorough understanding of the mechanisms driving sports injury occurrence.

The results highlight several critical factors influencing injury risk, encompassing both measurable training loads and athlete-reported perceptions. Elevated training demands–particularly high-intensity sprinting distances–can substantially increase musculoskeletal stress and, when paired with insufficient recovery, raise the likelihood of injury. At the same time, athlete perceptions of recovery quality and training success emerged as strong indicators of physical readiness and resilience; lower scores in these measures often reflect accumulated fatigue and compromised movement control. Variations in total running volume, session frequency, and strength training intensity were also found to influence these perceptions, thereby indirectly shaping injury risk. Practical applications of these insights include regulating sprinting distances and intensities to prevent acute overload, progressively adjusting total running volume and session frequency, and structuring strength training to maximize performance benefits without inducing overtraining. Athlete-reported measures of recovery and training success can serve as low-cost, real-time indicators for fine-tuning training plans before injuries occur.

From a policy perspective, integrating both objective load metrics and subjective perception measures into institutional injury surveillance systems would enhance early detection and prevention. Sports organizations could define evidence-based thresholds for key indicators, implement mandatory periodic monitoring, and foster a training culture that prioritizes recovery alongside performance goals. The quantile threshold framework used in this study also offers practical training load monitoring guidance. By determining safe ranges for load-related variables and coupling them with perceptual feedback, coaches can detect emerging imbalances between workload and recovery, enabling timely adjustments that maintain athletes in optimal performance zones while minimizing injury risk. Finally, the findings have important implications for recovery strategies. Structured recovery programs should address both physiological restoration–through rest intervals, active recovery, sleep optimization, and nutrition–and psychological readiness, by enhancing athletes confidence and perceived training success. A dual focus on physical and perceptual recovery can improve resilience to high-intensity demands and contribute to sustained performance with lower injury incidence.

This study proposes a quantile threshold function (QTF) that transforms continuous variables into ordinal variables. Although this method offers simplicity and effectiveness, it is essential to recognize that various alternative approaches exist for converting continuous variables into ordinal variables. Methods like decision tree binning and K-Means clustering can capture complex relationships between continuous variables and other features more precisely. However, they require higher computational power and suffer from poor theoretical interpretability. Future studies could explore hybrid discretization schemes that combine the advantages of multiple methods. For example, integrating statistical techniques with machine learning algorithms may yield more flexible and efficient discretization strategies. Investigating the impact of different discretization methods on subsequent tasks, such as predictive modeling or clustering, could also provide valuable insights for practical applications. By addressing these limitations and broadening the analytical perspective, future research is expected to explore optimal strategies for transforming continuous variables into ordinal categories, thereby advancing the development of more comprehensive data preprocessing frameworks and improving model performance.

This study examines a single-day subset of the dataset, which limits the generalizability of the findings. Future work will extend the analysis to weekly datasets and employ time-series methods to capture temporal dynamics better. Moreover, although the current study focuses on single interventions, it is crucial to acknowledge that, in real-world scenarios, any exogenous intervention can simultaneously influence multiple target variables. Therefore, predicting the impact of joint interventions on an outcome variable becomes a relevant consideration. The methods proposed by (47) can be readily extended to address multiple interventions. Consequently, under the latent Gaussian model, incorporating joint interventions–similar to the approach of (48) represents a natural progression within the latent space framework. In the main text, we also mentioned that some datasets contain both ordinal and non-ordinal data. Naturally, this leads us to consider the problem of Bayesian network learning with mixed data. In particular, one may obtain a dataset with both continuous and ordinal variables by first generating a Gaussian dataset according to a DAG structure and then discretizing some of the variables while keeping others continuous. A similar learning framework may be applicable in this context. Therefore, future research could explore using such learning frameworks for causal analysis of data.

## Data Availability

The datasets for this study can be found in the [shashwatwork/injury-prediction] repository on Kaggle https://www.kaggle.com/datasets/shashwatwork/injury-prediction-for-competitive-runners. Software in the form of R code, R code is available at [https://github.com/TaoXyjammy/OrdinalEffectsSport].
