# Supplementary material for: A causal inference method for athletic injuries based on quantile threshold functions and latent Gaussian DAG models
Source: Front Public Health. 2025 Sep 10;13:1647200. doi: 10.3389/fpubh.2025.1647200 (PMC12457826; doi:10.3389/fpubh.2025.1647200)
Supplement: Supplementary file 1 [file Supplementary_file_1.zip › Supplementary_Material.pdf]

Supplementary Material

1 SUPPLEMENTARY PROOF

Proof (for Proposition in the main text) The following provides the detailed proof of Proposition as stated in the main text. Since the proof is rather lengthy, it is presented here in the supplementary materials to avoid overloading the main article.

1.1 Proof of Proposition 1

PROOF.

$$\begin{aligned}\sum_{j=0}^{2}\mathbb{P}[g(x)=j] &= \mathbb{P}[g(x)=0]+\mathbb{P}[g(x)=1]+\mathbb{P}[g(x)=2]\\ &= \int_{-\infty}^{Q_i} f(x)dx + \int_{Q_i}^{Q_{i+1}} f(x)dx + \int_{Q_{i+1}}^{\infty} f(x)dx\\ &= \int_{-\infty}^{\infty} f(x)dx = 1\end{aligned}$$

1.2 Proof of Proposition 2

PROOF.

$$\begin{aligned}\sum_{j=0}^{n}\mathbb{P}[g(x)=j] &= \mathbb{P}[g(x)=0]+\mathbb{P}[g(x)=1]+\cdots+\mathbb{P}[g(x)=n]\\ &= \int_{-\infty}^{Q_i} f(x)dx + \int_{Q_i}^{Q_{i+1}} f(x)dx + \cdots + \int_{Q_{i+n-1}}^{\infty} f(x)dx\\ &= \int_{-\infty}^{\infty} f(x)dx = 1\end{aligned}$$

1.3 Proof of Proposition 3

PROOF.

$$\begin{aligned}\lim_{n\to\infty}\mathbb{P}\left[g(x)=n\right] &= \lim_{n\to\infty}\int_{Q_{i+n-1}}^{\infty} f(x)dx\\ &= 1-\lim_{n\to\infty} F\left(Q_{i+n-1}\right)\\ &= 1-\lim_{n\to\infty}\int_{-\infty}^{Q_{i+n-1}} f(x)dx\\ &= 1-\lim_{n\to\infty}\frac{i+n-1}{n+2} = 0 \quad (i \text{ is finite integer}).\end{aligned}$$

2 SUPPLEMENTARY TABLES

This file provides the ANOVA results for the three discretization methods (equal-width, equal-frequency, and k-means):

Table S1. ANOVA for the Three group Levels and Significance Testing based on the Equal-Width

	Group(Mean±SD)$_n$			F	p
**Ordinal**	**'0'**	**'1'**	**'2'**		
**Variable 2**	$5.752 \pm 5.887_{39739}$	$23.41 \pm 4.169_{2947}$	$42.743 \pm 4.951_{80}$	14311	0.001^{***}
**Variable 3**	$0.626 \pm 2.013_{42622}$	$18.17 \pm 2.78_{129}$	$33.346 \pm 5.12_{15}$	6825	0.001^{***}
**Variable 4**	$0.561 \pm 1.696_{42727}$	$20.331 \pm 3.309_{38}$	$48 \pm NA_{1}$	2963	0.001^{***}
**Variable 5**	$0.069 \pm 0.396_{42758}$	$15.5 \pm 1.568_{7}$	$40 \pm NA_{1}$	10350	0^{***}
**Variable 7**	$0.003 \pm 0.046_{21717}$	$0.542 \pm 0.085_{7282}$	$0.795 \pm 0.083_{13767}$	619371	0.001^{***}
**Variable 8**	$0.146 \pm 0.455_{42614}$	$4.283 \pm 0.826_{121}$	$8.02 \pm 0.882_{31}$	9518	0.001^{***}
**Variable 9**	$0.091 \pm 0.102_{28823}$	$0.489 \pm 0.096_{9916}$	$0.775 \pm 0.083_{4027}$	120860	0^{***}
**Variable 10**	$0.112 \pm 0.107_{33162}$	$0.462 \pm 0.086_{8813}$	$0.741 \pm 0.068_{791}$	51506	0.001^{***}

Table S2. ANOVA for the Three group Levels and Significance Testing based on the Equal-Frequency

	Group(Mean±SD)$_n$			F	p
**Ordinal**	**'0'**	**'1'**	**'2'**		
**Variable 2**	$0 \pm 0_{14256}$	$5.371 \pm 3.410_{14255}$	$15.744 \pm 5.273_{14255}$	69472	0.001^{***}
**Variable 3**	$0 \pm 0_{14256}$	$0 \pm 0_{14255}$	$2.074 \pm 3.639_{14255}$	4629.7	0.001^{***}
**Variable 4**	$0 \pm 0_{14256}$	$0 \pm 0_{14255}$	$1.739 \pm 2.798_{14255}$	5509.7	0.001^{***}
**Variable 5**	$0 \pm 0_{14256}$	$0 \pm 0_{14255}$	$0.219 \pm 0.818_{14255}$	1022	0.001^{***}
**Variable 7**	$-0.008 \pm 0.004_{14256}$	$0.267 \pm 0.266_{14255}$	$0.790 \pm 0.085_{14255}$	90105	0.001^{***}
**Variable 8**	$0 \pm 0_{14256}$	$0 \pm 0_{14255}$	$0.490 \pm 0.863_{14255}$	4596.9	0.001^{***}
**Variable 9**	$0.001 \pm 0.027_{14256}$	$0.176 \pm 0.063_{14255}$	$0.566 \pm 0.161_{14255}$	115477	0^{***}
**Variable 10**	$0.001 \pm 0.029_{14256}$	$0.167 \pm 0.034_{14255}$	$0.419 \pm 0.134_{14255}$	94205	0^{***}

Table S3. ANOVA for the Three group Levels and Significance Testing based on the K-Means

	Group(Mean±SD)$_n$			F	p
**Ordinal**	**'group 1'**	**'group 2'**	**'group 3'**		
**Variable 2**	$0.546 \pm 1.419_{20161}$	$20.937 \pm 5.244_{5324}$	$10.329 \pm 2.805_{17281}$	136700	0.001^{***}
**Variable 3**	$6.536 \pm 1.962_{3607}$	$0.045 \pm 0.329_{38905}$	$16.608 \pm 5.549_{254}$	168269	0.001^{***}
**Variable 4**	$0.045 \pm 0.255_{38427}$	$4.434 \pm 1.225_{3569}$	$9.392 \pm 3.372_{770}$	159352	0.001^{***}
**Variable 5**	$0.018 \pm 0.089_{41406}$	$10.210 \pm 5.505_{46}$	$1.443 \pm 0.866_{1314}$	58408	0.001^{***}
**Variable 7**	$0.524 \pm 0.094_{7160}$	$0.790 \pm 0.084_{14191}$	$-0.001 \pm 0.03_{21415}$	649340	0^{***}
**Variable 8**	$0.007 \pm 0.057_{38289}$	$1.301 \pm 0.487_{4140}$	$3.899 \pm 1.56_{337}$	121755	0.001^{***}
**Variable 9**	$0.369 \pm 0.095_{10875}$	$0.695 \pm 0.109_{7342}$	$0.059 \pm 0.075_{24549}$	164758	0^{***}
**Variable 10**	$0.002 \pm 0.031_{14330}$	$0.504 \pm 0.107_{8485}$	$0.203 \pm 0.066_{19951}$	144734	0^{***}

3 SUPPLEMENTARY FIGURES

The following is the probability density function graph for categorical variables.

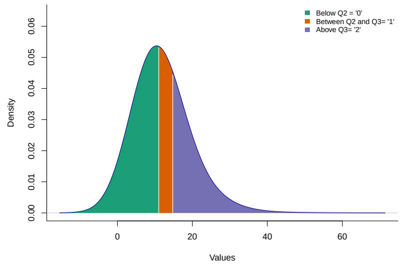

variable 2

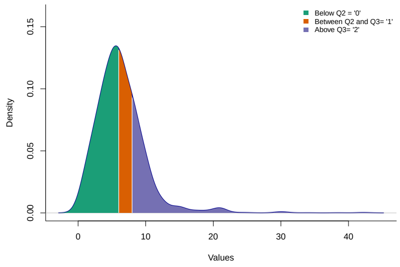

variable 3

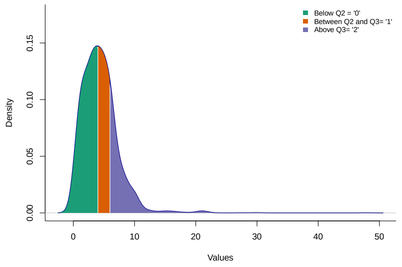

variable 4

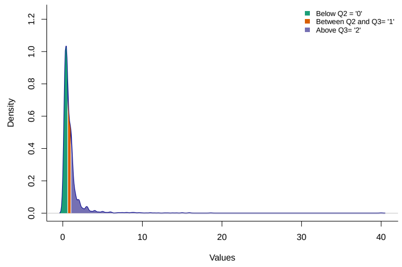

variable 5

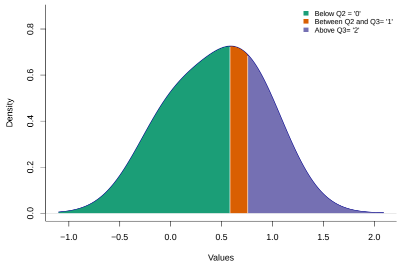

variable 7

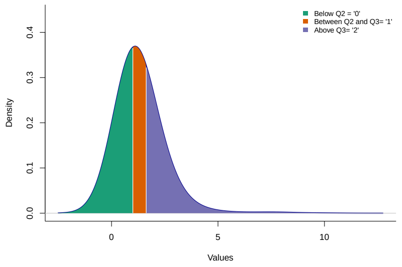

variable 8

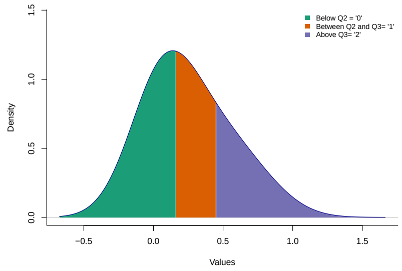

variable 9

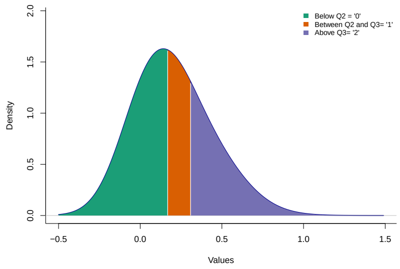

variable 10

Figure S1: The Probability Density Function and Quantile Ranges

This file supplements the heatmaps for the CPDAG adjacency matrices of the ordinal data in section 4.1 S2, as well as the ordinal causal effects of other variables S3.

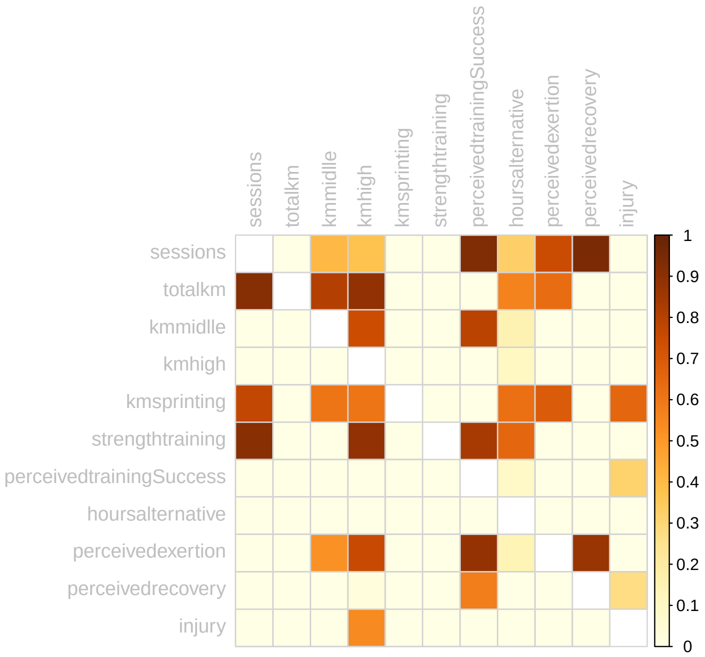

Figure S2: Heatmaps for the CPDAG adjacency matrices of the ordinal data of section 4.1.The darker the shade in the grid, the more frequently the corresponding directed edge occurs in the 500 Bootstrapped CPDAGs; where an indirect edge counts half for each direction.

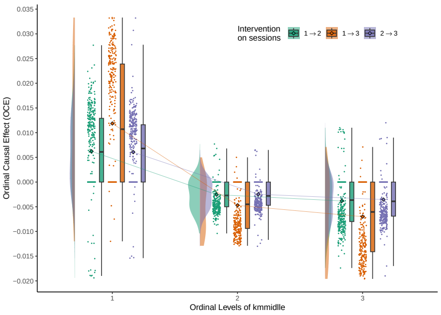

variable 1 → variable 3

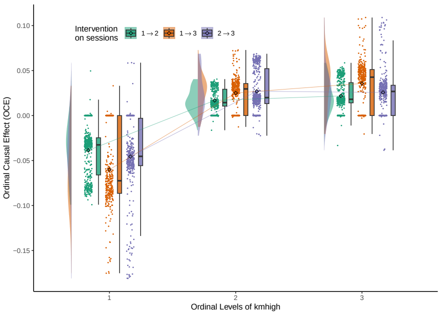

variable 1 → variable 4

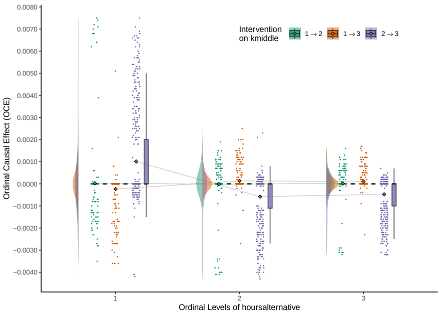

variable 3 → variable 8

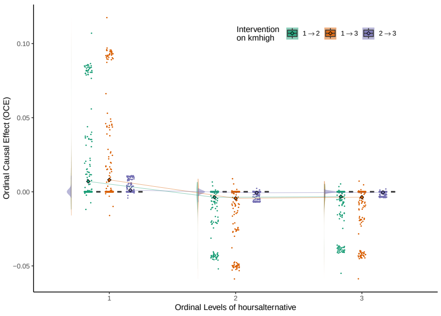

variable 4 → variable 8

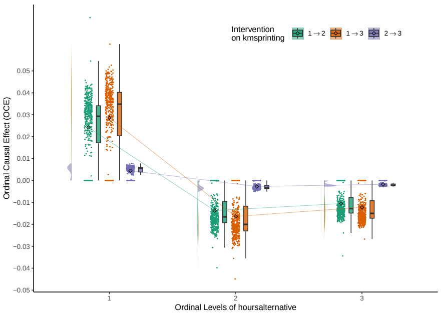

variable 5 → variable 8

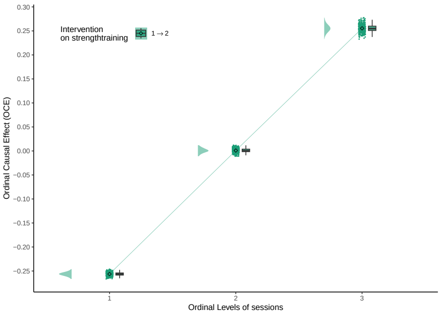

variable 6 → variable 1

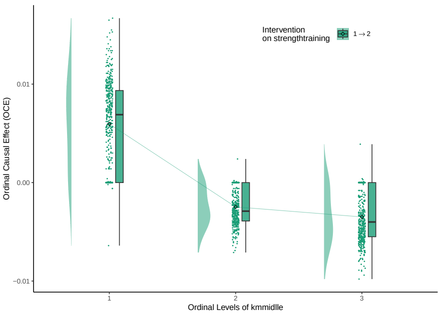

variable 6 → variable 3

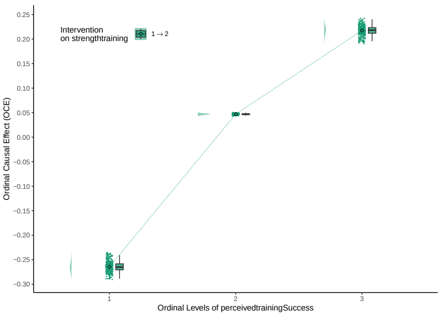

variable 6 → variable 7

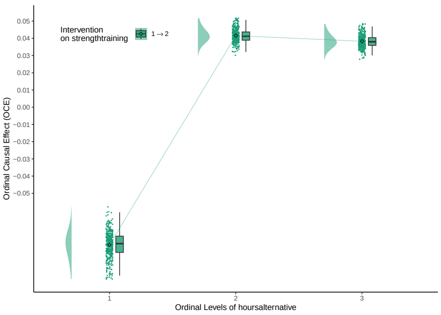

variable 6 → variable 8

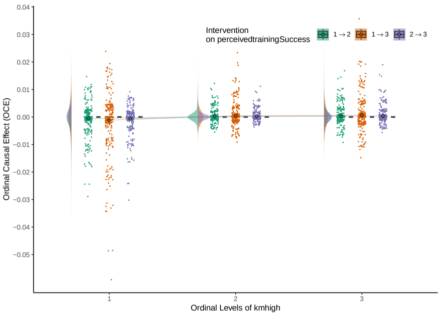

variable 7 → variable 4

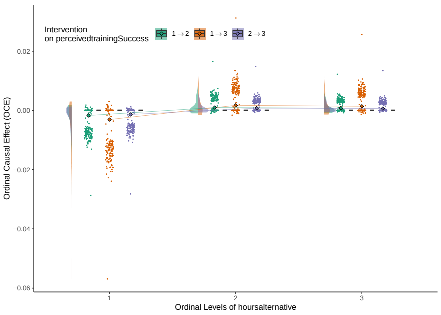

variable 7 → variable 8

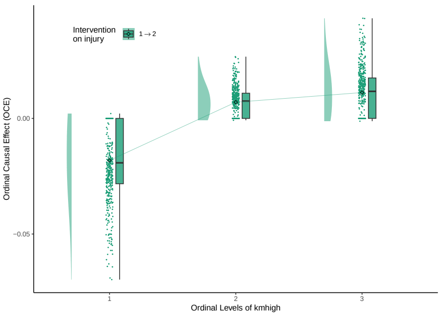

variable 11 → variable 4

Figure S3: Ordinal Causal Effect of intervention variable → outcome variable
